# Association of dietary variety with the risk for dementia: the Yabu cohort study

**DOI:** 10.1017/S1368980023000824

**Published:** 2023-11

**Authors:** Yuri Yokoyama, Yu Nofuji, Satoshi Seino, Takumi Abe, Hiroshi Murayama, Miki Narita, Shoji Shinkai, Akihiko Kitamura, Yoshinori Fujiwara

**Affiliations:** 1 Research Team for Social Participation and Healthy Aging, Tokyo Metropolitan Institute for Geriatrics and Gerontology, 35-2 Sakae, Itabashi, Tokyo 173-0015, Japan; 2 Department of Nutrition Sciences, Kagawa Nutrition University, Sakado City, Saitama, Japan; 3 Health Town Development Science Center, Yao City Health Center, Yao City, Osaka, Japan; 4 Tokyo Metropolitan Institute for Geriatrics and Gerontology, Itabashi, Tokyo, Japan

**Keywords:** Dietary variety, Disabling dementia, Dietary quality, Older adults

## Abstract

**Objective:**

The consumption of various foods is internationally recommended in healthy diet although the association between dietary variety and incident dementia is unknown. We aimed to examine the association between dietary variety and the incidence of disabling dementia in older Japanese adults.

**Design:**

We conducted a prospective cohort study. Dietary variety was assessed based on the Dietary Variety Score (DVS). DVS was assessed by counting the number of ten food components (meat, fish/shellfish, eggs, milk, soyabean products, green/yellow vegetables, potatoes, fruit, seaweed and fats/oils) that were consumed almost daily using a FFQ. Participants were categorised into low (0–2 points), middle (3–4 points) and high (5–10 points) groups based on the DVS. Data on newly diagnosed disabling dementia were retrieved from the public long-term care insurance database. Cox proportional hazards regression was used to estimate hazard ratios (HR) with 95 % CI.

**Setting:**

Yabu cohort study, Japan.

**Participants:**

A total of 4972 community-dwelling adults aged 65 years or older.

**Results:**

During the median follow-up of 6·8 years, 884 participants were newly diagnosed with disabling dementia. After adjusting for confounders, the multivariable-adjusted HR for incident disabling dementia was 0·82 (95 % CI, 0·69, 0·97) for participants in the highest DVS category compared with those in the lowest DVS category (*P*_for trend_ = 0·019).

**Conclusions:**

A higher dietary variety is associated with a reduced risk of disabling dementia in older Japanese adults. These results have potential implications for the development of effective public nutritional approaches to prevent dementia in older adults.

With the increase in global population ageing, dementia has become an important public health issue. Worldwide, more than 50 million people have dementia, and the number of those with dementia is expected to increase to 152 million by 2050^(1,2)^. Without an effective treatment for dementia, primary prevention has become a priority for public health, both to reduce the incidence and to delay the progression of dementia. According to the 2020 report of the Lancet Commission on Dementia Prevention, Intervention, and Care, targeting 12 modifiable risk factors (poor education, hypertension, hearing impairment, smoking, obesity, depression, physical inactivity, diabetes, limited social interaction, excessive alcohol consumption, traumatic brain injury and air pollution) over the life course might help prevent up to 40 % of dementia cases or to delay progression^(3)^.

Diet is a modifiable behavioural factor that can influence the risk of future dementia. Epidemiological studies have shown that some nutrients (e.g. folate, flavonoids, vitamin D and certain lipids) and food groups (e.g. seafood, vegetables and fruits as well as potentially moderate alcohol and caffeine consumption) are protective factors for cognitive outcomes in older people, although the results have been inconclusive^(4)^. Therefore, there is considerable interest in the role of dietary patterns and the effects of overall diet quality to help elucidate the complex biological interactions between dietary components.

Dietary variety (or diversity) has long been recognised as a key element of high-quality diets and is recommended in dietary guidelines because no single food can provide the appropriate amount of nutrients that are necessary to maintain optimal health^(5)^. Many dietary diversity indicators (DDI) have been developed to examine the association between DDIs and various health outcomes^(5)^. However, to our knowledge, few studies have examined the association between dietary variety and cognitive function compared with other health outcomes, and only three prospective studies have found a protective association of DDI with cognitive decline and hippocampal atrophy^(6–9)^. Although various cognitive outcomes (e.g. global cognitive function assessed by Mini-Mental State Examination and brain imaging biomarkers) have been investigated, no study has used the incidence of dementia as an outcome. Therefore, this study aimed to examine the association between dietary variety and incident disabling dementia in older Japanese adults.

## Methods

### Study design and participants

**Fig. 1:**
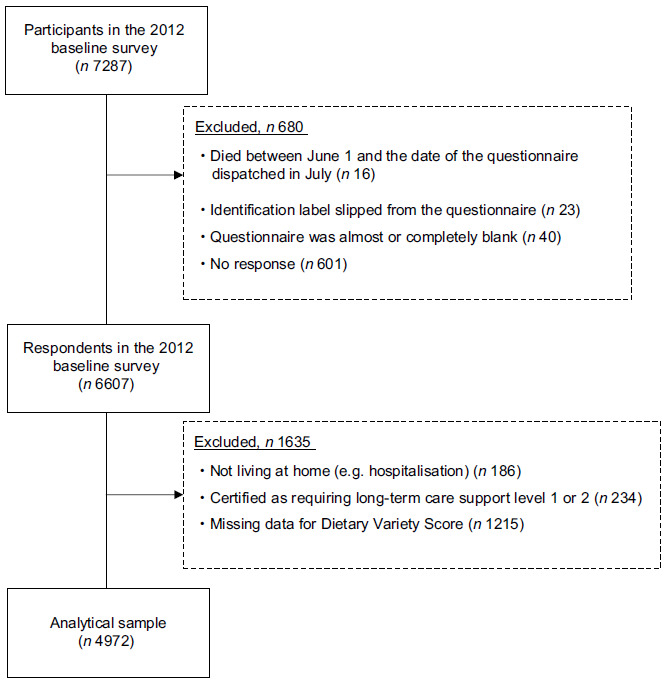
Flow chart of the participant enrolment in the study

This study is reported in accordance with the Strengthening the Reporting of Observational Studies in Epidemiology – Nutritional Epidemiology (STROBE-nut) checklist^(10)^. This study analysed data previously collected for the Yabu cohort study, and the details of the study design and participants have been reported previously^(11)^. The Yabu cohort study is a prospective study of community-dwelling individuals aged 65 years and more in Yabu, Hyogo Prefecture, Japan. The baseline survey was conducted between July and August 2012. Self-report questionnaires were distributed to all older adults aged 65 years or more in Yabu City who were not certified for long-term care insurance, care level 1–5 at baseline (*n* 7287) and were collected by mail. Of the 6607 participants who returned questionnaires, we excluded those who lived outside the city/ was admitted to hospital or residing in nursing homes (*n* 186); those who had already been certified for long-term care insurance, support level 1 or 2 because of a disability (*n* 234) and those with missing information on dietary variety (*n* 1215). Finally, the present study analysed data from 4972 participants (Fig. 1).

### Dietary variety

Dietary variety was assessed at the baseline survey using the Dietary Variety Score (DVS) that was developed by Kumagai et al.^(12)^. The DVS was assessed using a FFQ that determined the intake frequency of ten food groups (meat, fish/shellfish, eggs, milk, soyabean products, green/yellow vegetables, potatoes, fruit, seaweed and fats/oils) but not the portion size consumed for each group. The ten food groups constitute a large proportion of the daily main and side dishes in Japanese cuisine. The intake frequency of each food group was described using four options: (a) daily or almost daily (≥ 5 d a week), (b) once in 2 d, (c) once or twice a week and (d) hardly ever. Option (a) was assigned 1 point, whereas options (b) to (d), which represent intermittent consumption, were assigned 0 points. The DVS was calculated as the sum of the points. The total score ranged from 0 to 10, and higher scores indicated greater dietary variety. The study participants were categorised into low (0–2 points), middle (3–4 points) and high (5–10 points) DVS groups based on tertiles of the DVS. In sex-stratified analysis, sex-stratified tertiles of DVS were used (men: low (0–1 points), middle (2–3 points) and high (4–10 points); women: low (0–2 points), middle (3–4 points) and high (5–10 points)).

### Incident disabling dementia

Incident-disabling dementia was assessed according to the national long-term care insurance system, which provides national compulsory insurance for all individuals aged 40 years or more in Japan^(13–15)^. The certification of long-term needs was based on the assessment of applicants’ functional health status using a questionnaire that was developed by the Ministry of Health, Labour, and Welfare and based on the written opinion from the primary care physician.

In this study, disabling dementia was defined as a level II or higher on the Dementia Scale (classified into eight levels – 0, I, IIa, IIb, IIIa, IIIb, IV and M – by the degree of independence in the daily lives of individuals with dementia) that was reported by the primary care physicians for those who were certified as requiring long-term care, as described previously^(16–18)^. Level II refers to a state in which a subject at least manifests some symptoms, behaviours or communication difficulties that hinder their daily activities^(19)^. The Dementia Scale was validated in a previous study, which showed that the Dementia Scale is highly correlated with the Mini Mental State Examination Score (Spearman’s rank correlation coefficient −0·736)^(20)^. Furthermore, another study reported a sensitivity (95 % CI) of 73 % (65, 80 %) and specificity of 96 % (94, 97 %) for the Dementia Scale in comparison with clinical diagnoses by neuropsychiatrists (using a clinical interview as defined by the International Psychogeriatric Association)^(21)^.

### Covariates

The covariates used in the analyses were sex, age (65–69, 70–74, 75–79, 80–84 and ≥ 85 years), education (junior high school graduation, high school graduation, junior college/vocational college/college/graduate school graduation or other), living situation (living with others or alone), smoking habits (current, never or former), subjective poverty level (affluent, middle or poor), drinking habits (< 1 time/week or ≥ 1 times/week), exercise habits (< 1 time/week or ≥ 1 times/week), self-reported medical history (presence of hypertension, diabetes and stroke), BMI (< 18·5, 18·5–24·9 or ≥ 25 kg/m^2^), self-perceived chewing ability (good or poor), depressive symptoms and cognitive function (presence of cognitive complaints). Depressive symptoms were assessed using the fifteen-item Geriatric Depression Scale^(22,23)^. Cognitive function was assessed using three items from the Kihon Checklist-Cognitive Function Scale whose predictive validity has been previously confirmed^(24)^. We classified participants with at least one item as having a cognitive complaint. The BMI was calculated as the self-rated body weight (kg) divided by the self-rated height squared (m^2^). Covariates with missing data were assigned to the ‘missing’ category and included in the analysis as dummy variables to reduce selection bias.

### Statistical analysis

The characteristics of the study population, based on the DVS category, were compared using the *χ*^2^ test for categorical variables.

The person-years of follow-up were calculated for each participant from 1 July 2012 until the date of incident disabling dementia, migration from Yabu City, death or the end of the follow-up period (31 March 2019), whichever occurred first. In our analysis, data about participants who were lost to follow-up due to death or relocation before incident dementia were considered to be censored. The age- and sex-adjusted or multivariable-adjusted hazard ratios with 95 % CI were estimated using the Cox proportional hazards model. The multivariable model was adjusted for the following potential confounding variables: sex (only when analysing the sample as a whole), age, education, subjective poverty level, living situation, medical history (hypertension, diabetes and stroke), smoking habits, drinking habits, exercise habits, BMI, self-perceived chewing ability, depressive symptoms and cognitive function scores. The abovementioned potential confounders were chosen after reviewing previous findings^(25)^ that suggested a relationship between exposure and the outcome of interest. We additionally analysed the data stratified by sex.

To consider the possible influence of reverse causality, we conducted sensitivity analyses by excluding participants who experienced incident disabling dementia within the first 2 years of follow-up. Additionally, to examine whether the association would change if only individuals who had higher cognitive function at baseline were selected, another sensitivity analysis was conducted by excluding participants who scored ≥ 1 on the Kihon Checklist-Cognitive Function Scale.

A two-sided *P* value < 0·05 was considered indicative of statistical significance. Statistical analyses were performed using IBM SPSS Statistics version 23 (IBM Corp.).

## Results

During a median follow-up period of 6·8 years, 884 participants were newly diagnosed with disabling dementia, of whom 229 were newly diagnosed with disabling dementia in the first 2 years after the baseline survey.

The participant characteristics were stratified according to the DVS category (Table 1). Compared with participants with lower DVS, those with higher DVS were more likely to be older, be women, have completed many years of education, consider themselves affluent and exercise regularly. They were less likely to be current smokers or drinkers. Additionally, participants with higher DVS were less likely to have a history of hypertension, lower chewing ability, depressive symptoms and lower cognitive function.

**Table 1:** Participant characteristics stratified by the categories of the DVS

	DVS						
	Low (0–2 points)		Middle (3–4 points)		High (5–10 points)		*P* *
Number of participants	1997		1511		1464		
Women, *n* (%)	998	50·0	843	55·8	894	61·1	< 0·001
Age, *n* (%)							
65–69 years	477	23·9	340	22·5	255	17·4	< 0·001
70–74 years	520	26·0	337	22·3	306	20·9	
75–79 years	417	20·9	344	22·8	386	26·4	
80–84 years	329	16·5	284	18·8	319	21·8	
≥ 85 years	254	12·7	206	13·6	198	13·5	
Education, *n* (%)							
Junior high school graduation	1163	58·2	747	49·4	680	46·4	< 0·001
High school graduation	565	28·3	473	31·3	510	34·8	
Junior college/vocational college/college/graduate school graduation	165	8·3	188	12·4	202	13·8	
Other/missing	104	5·2	103	6·8	72	4·9	
Subjective poverty level, *n* (%)							
Affluent	142	7·1	156	10·3	190	13·0	< 0·001
Middle	1124	56·3	935	61·9	942	64·3	
Poor	671	33·6	363	24·0	292	19·9	
Missing data	60	3·0	57	3·8	40	2·7	
Living situation, *n* (%)							
Lives with other	1729	86·6	1324	87·6	1308	89·3	0·095
Lives alone	265	13·3	186	12·3	156	10·7	
Missing	3	0·2	1	0·1	0	0·0	
Smoking status, *n* (%)							
Never or former	1735	86·9	1387	91·8	1360	92·9	< 0·001
Current	210	10·5	104	6·9	63	4·3	
Missing	52	2·6	20	1·3	41	2·8	
Drinking habit, *n* (%)							
< 1 time/week	1263	63·2	1018	67·4	999	68·2	< 0·001
≥ 1 time/week	677	33·9	470	31·1	419	28·6	
Missing	57	2·9	23	1·5	46	3·1	
Exercise habit, *n* (%)							
< 1 time/week	987	49·4	589	39·0	500	34·2	< 0·001
≥ 1 time/week	970	48·6	884	58·5	910	62·2	
Missing	40	2·0	38	2·5	54	3·7	
Medical history, *n* (%)							
Hypertension	1114	55·8	790	52·3	751	51·3	0·016
Stroke	261	13·1	167	11·1	180	12·3	0·297
Diabetes mellitus	440	22·0	317	21·0	297	20·3	0·608
BMI (kg/m^2^)							
< 18·5	165	8·3	126	8·3	114	7·8	0·218
18·5–24·9	1352	67·7	1056	69·9	1050	71·7	
≥ 25	388	19·4	259	17·1	245	16·7	
Missing	92	4·6	70	4·6	55	3·8	
Self-perceived chewing ability, *n* (%)							
Good	1845	92·4	1451	96·0	1421	97·1	< 0·001
Poor	143	7·2	58	3·8	36	2·5	
Missing data	9	0·5	2	0·1	7	0·5	
Depressive symptoms							
Non-depressed (GDS < 6)	868	43·5	835	55·3	896	61·2	< 0·001
Depressed (GDS ≥ 6)	763	38·2	401	26·5	320	21·9	
Missing	366	18·3	275	18·2	248	16·9	
Cognitive function score, *n* (%)							
0	1172	58·7	957	63·3	941	64·3	0·003
≥ 1	782	39·2	522	34·5	484	33·1	
Missing	43	2·2	32	2·1	39	2·7	

* *P* values are based on the *χ*^2^ test. DVS, Dietary Variety Score.

Table 2 shows the association between DVS and incident disabling dementia. We found that a higher DVS score was inversely associated with the incident risk of disabling dementia. The multivariable-adjusted HR (95 % CI) of disabling dementia for the low, middle and high DVS categories were 1·00 (reference), 0·89 (0·75, 1·04) and 0·82 (0·69, 0·97), respectively (*P*_for trend_ = 0·019). The results based on sex-stratified data are also shown in Table 2. In men, after adjusting for covariates, participants with the highest DVS had a significantly lower risk of disabling dementia (HR, 0·76; 95 % CI, 0·58, 1·00) than those with the lowest DVS. In women, participants with the highest DVS had a lower risk of disabling dementia; however, this association disappeared after multivariable adjustment in model 2 (HR, 0·84; 95 % CI, 0·68, 1·05).

**Table 2:** Hazard ratios and 95 % confidence intervals for incident disabling dementia according to categories of the DVS

	DVS					
	Low	Middle		High		*P* for trend
		HR	95 % CI	HR	95 % CI	
All						
No. of participants	1997	1511		1464		
No. of events	374	260		250		
Model 1*	1 (reference)	0·79	0·67, 0·92	0·70	0·60, 0·83	< 0·001
Model 2†	1 (reference)	0·89	0·75, 1·04	0·82	0·69, 0·97	0·019
Men						
No. of participants	633	742		862		
No. of events	103	110		133		
Model 1*	1 (reference)	0·74	0·57, 0·97	0·65	0·50, 0·84	0·001
Model 2†	1 (reference)	0·81	0·61, 1·06	0·76	0·58, 1·00	0·054
Women						
No. of participants	998	843		894		
No. of events	218	160		160		
Model 1*	1 (reference)	0·83	0·68, 1·02	0·71	0·57, 0·87	0·001
Model 2†	1 (reference)	0·96	0·77, 1·19	0·84	0·68, 1·05	0·123

DVS, Dietary Variety Score.

* Model 1 was adjusted for age and sex (only for the sample as a whole).

† Model 2 was adjusted for the variables in Model 1 plus education, subjective poverty level, living situation, smoking habits, drinking habits, exercise habits, medical history (hypertension, diabetes and stroke), BMI, self-perceived chewing ability, depressive symptoms and cognitive function score.

To examine the possible reverse causality of the association between the DVS and incident disabling dementia, we analysed the association after excluding participants who developed incident disabling dementia in the first 2 years of follow-up, and the results were similar. The adjusted HR (95 % CI) of disabling dementia for the low, middle and high DVS categories were 1·00 (reference), 0·88 (0·72, 1·06) and 0·80 (0·66, 0·98), respectively (*P*_for trend_ = 0·026; data not shown). We also analysed the association after selecting 3070 participants who had better cognitive function (Kihon Checklist-Cognitive Function Scale = 0). Although the results derived from this sensitivity analysis were similar to those obtained from the main analysis, the association was no longer significant; the adjusted HRs (95 % CI) of disabling dementia for the low, middle and high DVS categories were 1·00 (reference), 0·98 (0·76, 1·28) and 0·87 (0·66, 1·14), respectively (*P*_for trend_ = 0·319; data not shown).

## Discussion

In this cohort study, we investigated the association between dietary variety and incident disabling dementia and found that dietary variety was significantly associated with a decreased risk of incident disabling dementia. To our knowledge, this is the first study to show an association between dietary variety and the risk of disabling dementia.

Several epidemiological studies have assessed the association between dietary variety (or diversity) and cognitive outcome. A cross-sectional study of older adults in China assessed the Dietary Diversity Score based on the intake frequencies of nine food groups (vegetables, fruits, legumes and its products, nuts, meat, eggs, fish, milk and dairy products and tea) and reported that a poor Dietary Diversity Score was significantly associated with worse cognitive function^(7)^. In a prospective study of older Japanese adults, dietary diversity was determined using the Quantitative Index for Dietary Diversity based on a 3-day dietary record and was inversely associated with cognitive decline^(6)^. Additionally, a recent prospective study in older Japanese adults reported that a higher dietary diversity, as assessed by the Quantitative Index for Dietary Diversity, was associated with less reduction in the hippocampal volume^(9)^. Although the measure of dietary variety used in previous studies differs from that in our study, the present study findings support the protective association between dietary variety and cognitive outcome reported in these previous studies.

In the present study, DVS was significantly associated with lower risk of disabling dementia in men. However, the association between DVS and disabling dementia was weak in women and disappeared after multivariable adjustment. This result indicates potential behavioural differences between the sexes and other factors that may influence the development of dementia in women. In addition, the difference in findings between men and women might be explained by the possibility that women are more likely to misreport intake than men. A previous study reported that sex is a contributing factor to misreporting of dietary intake^(26)^. Additionally, dietary variety may play a lesser role in the development of dementia in women than in men. Consistent with our study, previous studies suggest the possibility of differences between men and women in relation to the association between dietary pattern and cognitive impairment; however, the relevant evidence was inconsistent^(27,28)^. Further research is needed to explore sex-specific differences in the association between diet and dementia.

Specific nutrients are associated with cognitive outcome, and folate and vitamin B_12_ are required for the synthesis of methionine from homocysteine, which has neurotoxic effects, whereas vitamin B_6_ serves as a cofactor for enzymes that are involved in homocysteine metabolism^(29,30)^. Therefore, B vitamins, such as folate, vitamin B_12_ and vitamin B_6_, may have a protective effect on cognitive function by decreasing serum homocysteine levels. *n*-3 fatty acids (particularly docosahexanoic acid) play an indispensable role in neuronal membranes and are involved in maintaining optimal cell membrane structure and facilitating cell function and responses^(31,32)^. In addition, *n*-3 fatty acids could prevent dementia through their antithrombotic and anti-inflammatory properties^(25)^. Oxidative stress is hypothesised to be caused by reactive oxygen species, and oxidative stress or inadequate antioxidant defence is therefore involved in the pathogenesis and progression of cognitive impairment and dementia^(33)^. Thus, antioxidants, such as vitamins E and C, carotene and polyphenols, protect against oxidative stress by quenching singlet oxygen, scavenging free radicals and inhibiting liquid peroxidation^(34)^. Moreover, several trace minerals, such as Se, Zn and Cu, play important roles as cofactors in the antioxidant system^(4)^. As there is considerable decline in the food and energy intake with increasing age, eating various foods could be beneficial for ensuring sufficient intake of the abovementioned micronutrients and achieving dietary adequacy. In fact, the DVS includes components of food groups that are rich in antioxidants, B vitamins and *n*-3 fatty acids (e.g. fish, milk, vegetables and fruits). Moreover, our previous study reported that individuals with a higher DVS consumed greater amounts of micronutrients and had less inadequate intake of Mg, Zn and vitamin B_6_ than those with lower scores^(35)^. Thus, the protective association between dietary variety and dementia may be ascribed to the combined effect of these nutrients on dementia.

The strengths of the present study include the prospective design, robust results of sensitivity analysis and adjustment for known and suspected risk factors for dementia. Moreover, the DVS is a simple measure of diet quality and can be assessed by individuals without nutritional expertise using a short food-based questionnaire. Previous studies have shown that it is possible to improve the DVS through self-checks using check sheets and nutrition education^(36,37)^. Therefore, the present findings of a protective association between dietary variety and disabling dementia are expected to guide the development of effective approaches for public nutrition. However, our study has several limitations. First, we assessed incident dementia using the long-term care insurance system rather than a clinical diagnosis of dementia, which may lead to a misclassification of incident dementia. Furthermore, because data from the long-term care insurance system did not include the type of dementia, such as Alzheimer’s dementia or vascular dementia, our analysis was limited to all-cause dementia. Further studies are required to examine the association between the DVS and specific types of dementia. Second, a DVS based on a ten-item FFQ may not be fully valid. DVS was evaluated based only on the number of different food groups consumed every day, without considering the minimum intake of the food groups. Thus, it is unclear whether the association of dietary variety with disabling dementia is independent of the amount of each food group consumed. Although we confirmed that intake assessed by 3-day dietary records was positively associated with consumption frequencies for the ten food groups in our preliminary analysis of another cohort (median Goodman and Kruskal’s gamma, 0·34), further studies that include quantitative evaluation of food intakes should be conducted. Third, the DVS was measured at only baseline. Although we could not evaluate changes during follow-up, our previous study identified three trajectory patterns (high, middle and low) in the DVS for the age period 65 years through 90 years; these trajectory patterns remained stable^(38)^. Fourth, reverse causation remains a concern because of the relatively short follow-up period (median, 6·8 years). Although we confirmed that the result did not change in the sensitivity analysis in which patients who developed incident disabling dementia in the first 2 years of follow-up were excluded, the association was no longer significant due, in part, to the reduced sample size and number of events investigated in another sensitivity analysis that included participants who had better cognitive function. To overcome this limitation, a cohort study with a longer follow-up and larger sample size is needed. Fifth, the DVS that was derived from self-administered mail surveys in this study was lower than that recorded in health check-up surveys^(12,39)^. We categorised DVS according to the distribution of the study population; thus, the group with the highest DVS might not necessarily reflect the optimal DVS. Therefore, further research involving samples representative of older Japanese adults is needed to determine the optimal cut-off value of DVS. Furthermore, because this study was conducted at only one site in Japan, it is possible that our results have limited generalisability. Finally, we were unable to adjust for energy intake because we could not quantitatively assess the dietary intake. Although we adjusted for several key factors of energy intake, such as sex, age and BMI, we could not completely exclude the possibility of unmeasured and residual confounding factors, including energy intake. Considering the possible confounding effect of energy intake on the association between DVS and depression^(40)^, additional adjustment for energy intake may have attenuated the observed associations.

In conclusion, this study showed that dietary variety is associated with a decreased risk of incident disabling dementia. Our findings suggest that dietary variety is important for preventing or delaying the onset of dementia.
